# A rare case of keratitis caused by *Scedosporium aurantiacum*: a case report and review highlight the need for early microbiological diagnosis and treatment

**DOI:** 10.3389/fmed.2026.1960357

**Published:** 2026-10-21

**Authors:** Jana Štanclová, Rudolf Kukla, Petra Česáková, Kateřina Nouzovská, Radka Bolehovská, Lenka Ryšková, Miroslav Fajfr, Marie Vajrychová, Pavel Boštík

**Affiliations:** 1 Institute of Clinical Microbiology, University Hospital Hradec Kralove, Hradec Králové, Czechia; 2 Institute of Clinical Microbiology, Faculty of Medicine in Hradec Kralove, Charles University, Hradec Králové, Czechia; 3 Ophthalmology Department, University Hospital Hradec Kralove, Hradec Králové, Czechia; 4 Faculty of Medicine in Hradec Kralove, Charles University, Hradec Králové, Czechia; 5 Biomedical Research Center, University Hospital Hradec Kralove, Hradec Králové, Czechia

**Keywords:** keratitis, MALDI-TOF MS, microbial culture, *Scedosporium aurantiacum*, surgery

## Abstract

*Scedosporium aurantiacum* is a fungus that is commonly present in the environment of Australia and Japan but has also been reported from some European regions. The diagnosis of scedosporiosis is dependent on culture despite its slow growth, with the subsequent identification using MALDI-TOF MS or sequencing of the specific genomic region. This study describes a very rare case of the infectious keratitis caused by *S. aurantiacum* and probably the first such case reported in Europe. The patient was an 80-year-old woman, referred to the Ophthalmology Department of the University Hospital in Hradec Kralove with a painful corneal ulcer of her right eye, which was deteriorating despite a previous topical and oral antibiotic treatment elsewhere. Her medical history included unspecified bilateral lacrimal drainage system surgery and a partial lateral tarsorrhaphy of her right eyelids due to a keratitis caused by lagophthalmos. Three days after severing of the tarsorrhaphy, ocular pain appeared and corneal ulcer developed, accompanied by progressive inflammation in the anterior chamber. A microbiological culture analysis of conjunctival swabs and corneal scraping yielded colonies of filamentous fungi, which was subsequently identified by MALDI-TOF MS as *Scedosporium aurantiacum* with a score consistent with species-level identification. Based on these findings, treatment with topical and oral voriconazole was initiated. Ocular infections caused by *S. aurantiacum* are extremely rare, but this species appears to be an emerging pathogen for infectious keratitis. To date, only three similar cases have been reported and well described worldwide. The infection is often indistinguishable from other fungal infections, and the therapy depends on the speedy identification of fungal species and its susceptibility to antifungals.

## 1 Introduction

Species of *Scedosporium* are filamentous fungi widely present in the environment, mostly in soils, sewers, standing water sources, or even in potted plants in hospitals (1, 2). These fungi have been recognized as emerging opportunistic pathogens mostly for immunocompromised but also for immunocompetent patients. In the latter, *Scedosporium* has been reported to cause trauma-associated infections. In immunocompromised patients, *Scedosporium* has been shown to infect various organs, including lung, central nervous system, bone, joint, skin, and subcutaneous tissue, causing rarely fatal invasive disease. Thus, the rate of mortality of invasive *Scedosporium* infection in patients with underlying malignancies has been reported to be as high as 55%, and even a higher mortality rate (75%) has been described in HSCT recipients (3). Reports from some countries show *Scedosporium* species as the second most common filamentous fungi identified in the airways of patients with cystic fibrosis (1, 2, 4).

The taxonomy of the genus *Scedosporium* is continuously evolving. Currently, more than 20 accepted species are recognized, of which *S. apiospermum*, *S. aurantiacum*, and *S. boydii* are among the most clinically relevant human pathogens (5). Antifungal therapy is often difficult to administer because species of *Scedosporium* are generally resistant to amphotericin B, 5-flucytosine, and several triazole antifungals, particularly fluconazole and itraconazole (5, 6). Low median MIC values have been reported for voriconazole (0.5 mg/L) and posaconazole (1.5 mg/L) (3), although the activity of posaconazole may vary among species and isolates. Voriconazole has been reported to exhibit the most potent *in vitro* activity against *S. aurantiacum* (4, 7) and ECMM/ISHAM/ASM guidelines recommend voriconazole-based therapy as the first-line treatment for invasive scedosporiosis (8). However, evidence for *Scedosporium* spp. or even *S. aurantiacum* keratitis treatment remains very limited. The newly developed antifungal olorofim demonstrated the lowest median MIC (0.0039 mg/L) among all antifungals tested by Seidel et al., suggesting its potential as a future therapeutic option for *Scedosporium* infections (3). More recently, another novel antifungal drug fosmanogepix displayed good *in vitro* activity against *Scedosporium* spp (9).

## 2 Case presentation

In July 2019, an 80-year-old non-smoking, non-alcohol-consuming woman was referred to the Department of Ophthalmology at University Hospital Hradec Kralove with a painful corneal ulcer in her right eye that had deteriorated despite topical and systemic antibiotic therapy. Her medical history included type 2 diabetes mellitus treated with oral antidiabetic medication and insulin. Furthermore, arterial hypertension accompanied by a previous heart attack and stroke resulting in right-sided hemiparesis was listed. She had undergone unspecified bilateral lacrimal drainage system surgery in the second decade of her life.

Nine months prior to presentation, the patient had developed peripheral facial nerve palsy with subsequent exposure keratitis secondary to lagophthalmos, for which a partial lateral tarsorrhaphy of the right eyelids was performed at another institution. Three days after severing of the tarsorrhaphy at the same institution, she developed ocular pain, and a corneal ulcer was diagnosed. Despite treatment with 0.3% of topical ofloxacin hourly, 3 mg/g of topical ofloxacin at bedtime, and 500 mg of oral ciprofloxacin twice daily for 3 days, the corneal findings progressively worsened, accompanied by increasing anterior chamber (AC) inflammation and hypopyon formation. Therefore, the patient was referred to our department, 10 days after severing of the tarsorrhaphy.

Visual acuity in her right eye was reduced to light perception. A slit-lamp examination revealed marked conjunctival hyperemia with mucous discharge and a central extensive deep corneal ulcer extending to the posterior stroma, associated with dense white stromal infiltration and overlying epithelial defect. The result of the Seidel test was negative. The corneal surface was irregular with superficial and deep corneal neovascularization in inferior quadrants. The AC showed 3+ cells (SUN grading scale), with a 4-mm hypopyon. A dense inflammatory membrane had formed, obscuring the pupil and extending into the iridocorneal angle, resulting in elevated intraocular pressure (IOP, measured by digital palpation). A B-scan ultrasonography did not show signs of endophthalmitis and the retina was attached. The left eye was unremarkable, with no signs of ocular pathology.

On the day of admission, conjunctival swabs from both eyes and corneal scraping from the right eye were obtained for a microbiological examination. The differential diagnosis included initially severe bacterial keratitis. In the operating theater, the AC washout was done, followed by an intracameral administration of 0.1 mL of cefuroxime (1 mg). Because of severe corneal stromal melting, an amniotic membrane transplantation was performed using a combined multilayered graft-and-single layer patch technique, followed by the application of a bandage contact lens. On the same day, oral ciprofloxacin was withdrawn and empirical intravenous antibiotic therapy with ceftazidime (2 g every 8 h) and vancomycin (750 mg every 12 h) was initiated, with dosing adjusted according to renal function. The clinical course and timing of the interventions are summarized in Figure 1.

**Figure 1:**
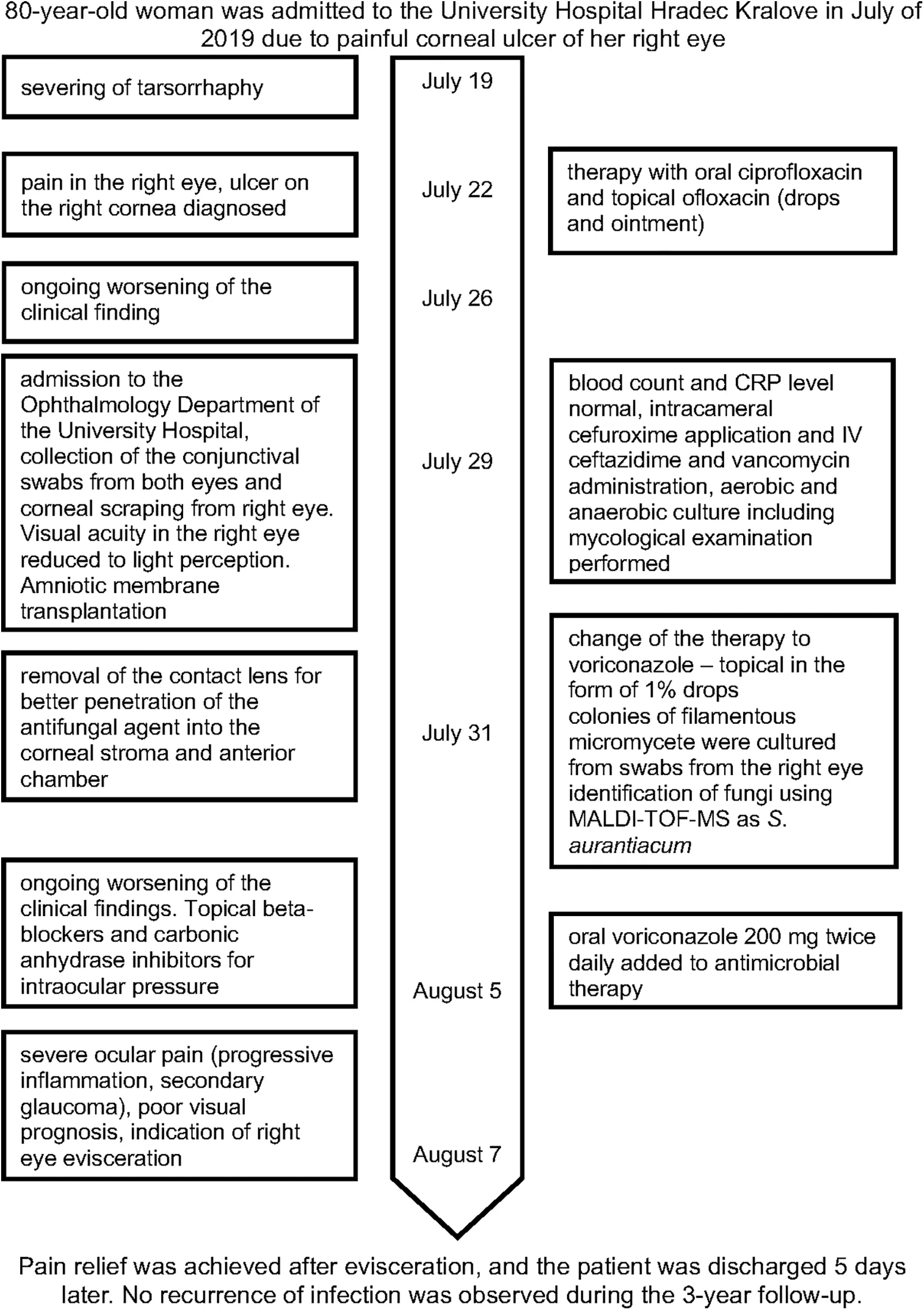
A timeline of the clinical course.

The ocular samples were sent for a microbiological examination, where cultures were performed on blood agar (BA), MacConkey agar (OXOID, UK), chocolate agar (bioMérieux, France), and Sabouraud glucose agar (SGA) (LMS, Czechia) aerobically for a prolonged period of 4 days at 37 °C and on Schaedler agar (bioMérieux, France) under anaerobic condition for 4 days at 37 °C. The colonies of filamentous micromycete appeared on BA and SGA from the swabs/samples of the right conjunctiva and cornea in 48 h in the range of 17–24 CFU. After a subculture, the colonies reached approximately 25–30 mm in diameter within 5 days. The colonies were dense, woolly to floccose, and grayish white on the obverse. The reverse was brown to orange, with a well-developed yellow marginal zone and slight diffusion of yellow pigment into the agar (Figures 2C,D). A direct microscopic examination of the colonies revealed abundant, hyaline, septate, and branching hyphae forming a loose aerial mycelium (Figures 2A,B). The culture of the swab from the left conjunctiva was mycologically negative. Native preparation revealed undifferentiated mycotic filaments with predominantly ovoid to ellipsoidal, occasionally short cylindrical conidia (Figure 3). The fungal isolate was identified as *Scedosporium aurantiacum* by MALDI-TOF MS using a microflex LT mass spectrometer. Mass spectra were analyzed using MALDI Biotyper Compass Explorer software version 3.1 and compared against the MBT Compass Reference Library Version 8.0, 7854 MSP (Bruker Daltonics, Bremen, Germany). Measurements were performed in triplicate. The best spectral scores obtained in the three measurements were 1.764, 1.937, and 2.045, respectively. The highest score (2.045) indicated reliable species-level identification. Score interpretation criteria were applied according to the manufacturer's recommendations and Barker et al. (10), i.e., score ≥2.0: reliable species-level identification; score ≥1.7 and <2.0: reliable genus-level identification; and score <1.7: unreliable identification. Fungal sample pretreatment was performed using the Mycelium Transfer (MyT) procedure according to the manufacturer's instructions. A sterile wooden applicator stick was dipped into a droplet of formic acid, and the moistened tip was used to collect the front mycelium of *S. aurantiacum*. The harvested mycelium was then smeared directly into the formic acid droplet on the MALDI target plate. After air-drying, 1 µL of *α*-cyano-4-hydroxycinnamic acid matrix solution was applied and allowed to dry completely before analysis. Species identification was supported by the production of a yellow diffusible pigment, a phenotypic characteristic reported for *S. aurantiacum* and not typically observed in other clinically relevant members of the genus *Scedosporium.* Microscopic morphology including ovoid to ellipsoidal was also consistent with *S. aurantiacum.* However, these features are not sufficiently specific for reliable differentiation among species of *Scedosporium* (11). These laboratory findings, along with the failure of antibiotic treatment, quickly led to the diagnosis of fungal keratitis.

**Figure 2:**
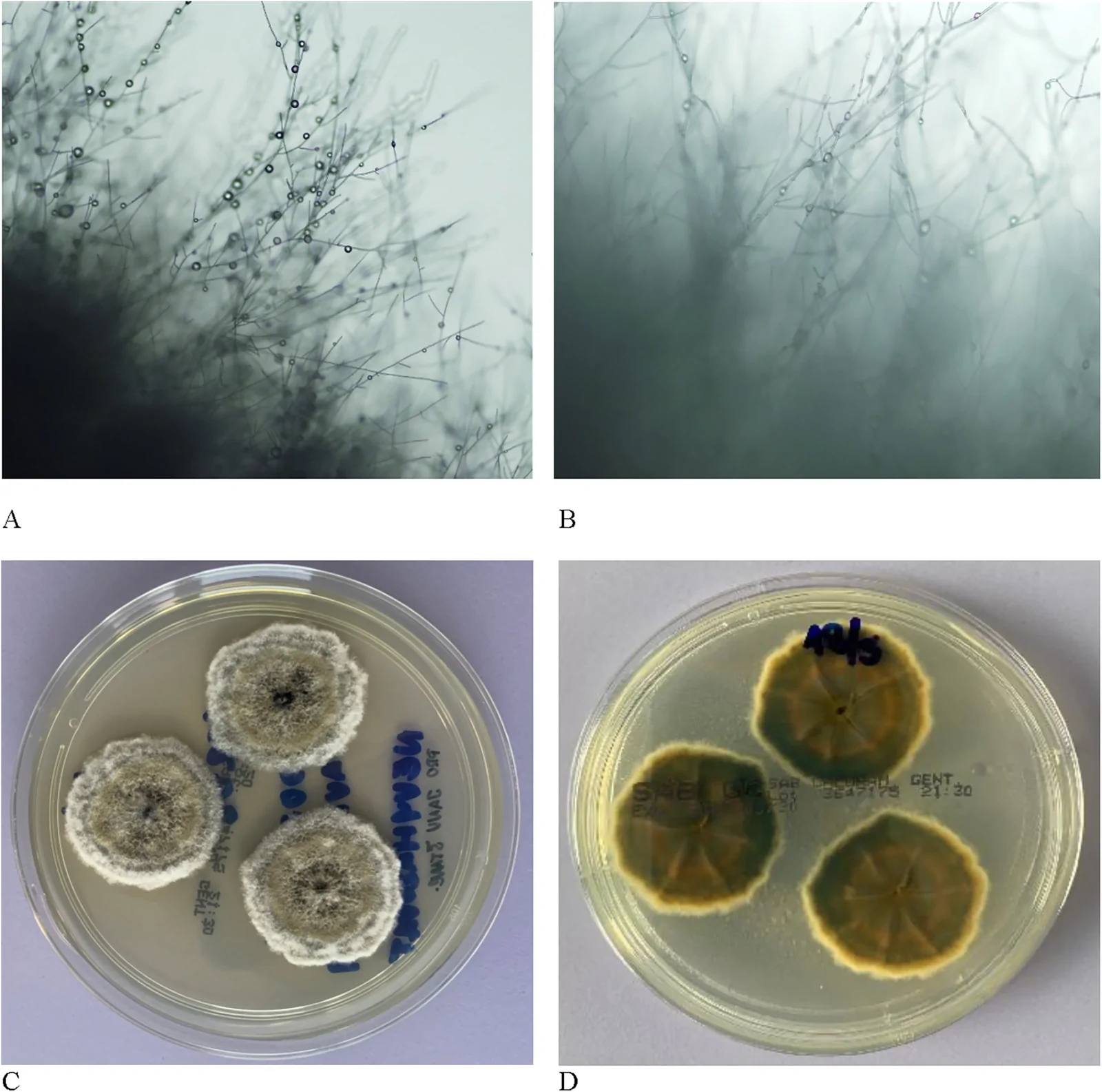
*S. aurantiacum* colonies on Sabouraud glucose agar incubated at 37 °C for 4 days. **(A)** and **(B)** Microscopic morphology of the colonies; **(A)** magnification 100× and **(B)** magnification 200×. **(C)** and **(D)** macroscopic morphology; **(C)** front view and **(D)** reverse view. After subculture, *S. aurantiacum* colonies reached approximately 25–30 mm in diameter within 5 days at 37 °C. The colonies were dense, woolly to floccose, and grayish white on the obverse **(C)**. The reverse was brown to orange, with a well-developed yellow marginal zone and slight diffusion of yellow pigment into the agar **(D)**. A direct microscopic examination of the colonies revealed abundant, hyaline, septate, and branching hyphae forming a loose aerial mycelium. **(C)** (magnification 100×) and **(D)** (magnification 200×).

**Figure 3:**
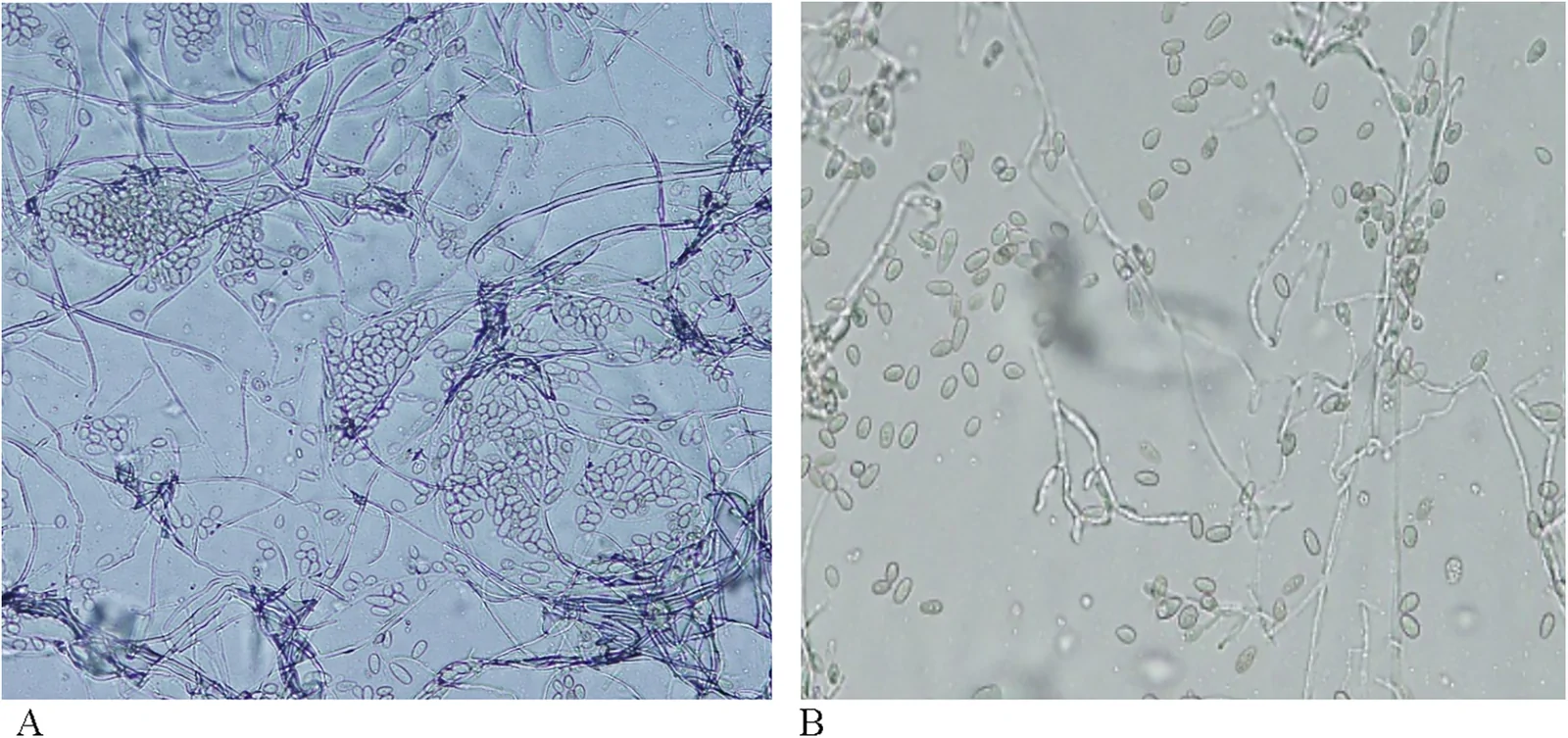
A microscopic morphology of *S. aurantiacum*. **(A)** impression preparation and **(B)** native preparation with Lugol's solution, magnification 400×. Septate hyphae with long conidiophores bearing conidia. Conidia are predominantly ovoid to ellipsoidal and occasionally short cylindrical.

Susceptibility to antifungal agents (Table 1) was determined by a disk diffusion method (BioVendor, Czechia) on Mueller Hinton agar supplemented by 2% of glucose with methylene blue (LabMediaServis, Czechia), an E-test assay for voriconazole (BioVendor, Czechia), and the broth microdilution non-reference method, Sensititre YeastOne (Trek Diagnostic Systems, USA). Although Sensititre YeastOne is primarily intended for susceptibility testing of *Candida* spp (12), it reported values exceeding 90% agreement between the CLSI M38-A2 method and Sensititre YeastOne for most antifungal agents tested against non-*Aspergillus* filamentous fungi.

**Table 1:** Susceptibility of *S. aurantiacum* to selected antifungals determined by the disk diffusion method.

Antifungal	Inhibition zone (mm)	Interpretation
Amphotericin B	13	N/A
Fluconazole	20	N/A
Posaconazole	15	N/A
Voriconazole	32	N/A
Clotrimazole	22	N/A
Terbinafine	9	N/A
Nystatin	12	N/A
Caspofungin	6	N/A

A suspension for antifungal susceptibility testing was prepared from a well-sporulating strain. Conidia were aseptically collected using a sterile cotton swab and suspended in physiological saline containing 0.05% Tween 20. After allowing heavy particles to settle, the conidial suspension was collected from the supernatant. The inoculum density was adjusted to 0.5–5 × 10^6^ CFU/mL using a densitometer, and the final inoculum concentration in each test well was 0.5–5 × 10^4^ CFU/mL (Biosan, Latvia). The procedure was performed according to the CLSI M38 guidelines for filamentous fungi (3rd edition). The quality-control organisms used in our laboratory include *Candida albicans* ATCC 90028, *Candida krusei* ATCC 6258, and *Candida parapsilosis* ATCC 22019. The media were incubated aerobically at 37 °C for 48 h. For azoles and amphotericin B, the MIC endpoint was determined as the concentration reducing approximately 80% and 100% of growth, respectively, compared with growth control. The minimum inhibitory concentration of amphotericin B, 5-flucytosine, posaconazole, voriconazole, itraconazole, and fluconazole for the isolate determined at 48 h were 2, >64; 0.12; 0.38; 0.12, and 8 mg/L, respectively (Table 2). Susceptibility testing for echinocandins was performed, but the results were excluded because minimum effective concentrations (MECs), rather than MICs, are the recommended endpoint for filamentous fungi and were not determined. The results of the disk diffusion method are presented in Table 1. Despite the absence of established clinical breakpoints, the disk diffusion findings were consistent with the MIC results. Thus, a large inhibition zone around the voriconazole disk corresponded to a relatively low MIC value. It should be noted that the clinical significance of voriconazole remains uncertain because of the non-standardized methods and absence of species-specific clinical breakpoints.

**Table 2:** A comparison of previously reported cases of ocular infections caused by *S. aurantiacum.*

Author information	Kim et al. (2)	Li et al. (14)	Carnovale et al. (1)	Present study
Antifungal	MIC (µg/mL)/the method used for MIC determination			
Amphotericin B	16 CLSI	NR	8 CLSI	2/SENS
Fluconazole	—	NR	—	8/SENS
Itraconazole	16 CLSI	NR	0.5 CLSI	0.12/SENS
Posaconazole	2 CLSI	NR	0.5 CLSI	0.12/SENS
Voriconazole	1 CLSI	NR	0.25 CLSI	0.38/ETEST
Isavuconazole	—	NR	2 CLSI	NR
Caspofungin	8 CLSI	NR	NR	NR
Micafungin	8 CLSI	NR	NR	NR
5-fluorocytosine	NR	NR	NR	>64 SE
Patient age, gender	70 years, woman	67 years, female	4 years, female	80 years, female
Predisposing factors	Mild eye injury due to soil exposition	Previous pterygium surgery with conjunctival autograft	Penetrating trauma by kitchen instrument	Lateral tarsorrhaphy of the right eyelids
Type of ocular infection	Infectious scleritis with corneal ulcer	Infectious scleritis with vitritis	Post-traumatic endophthalmitis	Keratitis
Identification/time to diagnosis	Culture and sequencing of the ITS region and the 26S rRNA gene/12 days	Culture/6 weeks	Culture and sequencing of the *β*-tubulin gene/15 days	Culture and MALDI-TOF MS/9 days
Country/region	Republic of Korea	Australia, Queensland	Argentina	Czech Republic
Targeted therapy	Topical voriconazole and amphotericin B, IV voriconazole	Topical + intravitreal + PO voriconazole	Voriconazole	Topical + oral voriconazole
Surgical intervention	Evisceration and enucleation	Enucleation	Surgery for corneal trauma	Evisceration
Follow-up duration	NR	NR	NR	3 years
Final visual outcome	Loss of eye	Loss of eye	A favorable clinical outcome	Loss of vision

CLSI, CLSI Standards M38 Reference method for broth dilution antifungal susceptibility testing of filamentous fungi ETEST, E-test, SENS, Sensititre YeastOne, NR, not reported.

Species of *Scedosporium* are commonly resistant to multiple antifungal agents such as 5-flucytosine, amphotericin B, and a majority of triazoles, e.g., fluconazole and itraconazole. They are also characterized by a reduced susceptibility to echinocandins, especially to caspofungin and anidulafungin. According to European guidelines, voriconazole is recommended for the treatment of infections caused by *Scedosporium* spp (1). CLSI has not yet established clinical sensitivity cutoff values for this genus. Based on the susceptibility results (Table 2), intravenous antibiotics were stopped and topical voriconazole 1% eye drops were initiated hourly. To facilitate penetration of the antifungal agent into the cornea and anterior chamber, the bandage contact lens was removed. However, because of continued clinical deterioration, systemic therapy with oral voriconazole (200 mg twice daily) was subsequently added. Intraocular pressure was managed with topical beta-blockers and carbonic anhydrase inhibitors, both administered twice daily, in combination with oral acetazolamide (250 mg three times daily). Despite intensive antifungal and IOP-lowering therapy, as well as analgesic treatment with oral metamizole (500 mg every 6 h as needed), severe ocular pain persisted. Visual acuity did not improve during the course of treatment and remained at the level of light perception. Given the intractable pain, poor visual prognosis, and absence of clinical evidence of scleral or extraocular involvement, evisceration was discussed with the patient and subsequently performed under general anesthesia. Postoperatively, all antimicrobial therapy was discontinued except for topical antibiotic ointment (neomycin sulfate and zinc bacitracin), administered five times daily. The patient experienced pain relief, ocular discomfort gradually reduced, and she was discharged 5 days later. The patient was satisfied with the outcome, and no recurrence of infection or other symptoms were observed during the 3-year follow-up. A histopathological examination of the removed ocular tissue confirmed the presence of fungal hyphae within the corneal tissue, demonstrated by Grocott's and periodic acid–Schiff (PAS) staining, accompanied by neutrophilic infiltration. No culture was performed on the eviscerated ocular tissue.

## 3 Discussion

This case report describes a rare ocular infection caused *by S. aurantiacum* and, likely, the first such case described in Europe. So far, only three cases of eye infections caused by *S. aurantiacum* have been well described worldwide (summarized in Table 2). A literature search was conducted on 1 September 2026, to identify previously published ocular infections caused by *S. aurantiacum*. PubMed and Google Scholar databases were searched using the terms “*Scedosporium aurantiacum*”, “ocular infection”, “keratitis”, “scleritis”, “endophthalmitis”, and “eye infection”. Only those publications in which at least the title or abstract was available in English were considered. In addition, the reference lists of relevant publications were manually screened to identify additional reports. Only cases with confirmed identification of *S. aurantiacum* were included. An additional ocular case was reported by Heath et al. in Australia (13). However, because no further clinical details were available, this report was consequently excluded from the case report analysis.

Kim et al. (2) described a case of a 70-year-old immunocompetent woman without diabetes from South Korea. The patient was admitted to a hospital 10 days after a mild eye injury contaminated by soil. Conjunctival infection, a large stromal infiltration, corneal lesion, and hypopyon were diagnosed. A microscopy of corneal scrapings showed fungal hyphae and the subsequent culture revealed *S. aurantiacum* after 5 days of incubation. Additional identification of *S. aurantiacum* was done by performing a sequence analysis of the ITS region. The initial combination therapy of topical moxifloxacin, fluconazole, and amphotericin B with IV fluconazole was changed to topical and intravenous voriconazole, topical moxifloxacin, and amphotericin B. After 1 month, the corneal lesion stabilized, but subsequently, 3 months later, the corneal stromal infiltration, hypopyon, and corneal melting significantly worsened and corneal perforation occurred. Therefore, evisceration of the eye with silicone ball implantation was performed to solve the infectious complications. Because of the postoperative infectious complications, an enucleation was performed and the patient was treated with topical voriconazole and amphotericin B combined with IV voriconazole. After 9 weeks of the antifungal therapy, the infection was cured.

The second case from Australia was reported by Li et al. (14). A 67-year-old woman presented with a discomfort in the left eye 6 months after pterygium excision with a medical history of Graves’ disease. An autoimmune scleritis was presumed and, therefore, dexamethasone drops and oral prednisolone were administered. Four days later, the patient presented with complications leading to an antimicrobial therapy, including the intravitreal application of voriconazole, vancomycin, and ceftazidime together with topical gentamicin, vancomycin, and voriconazole. A pars plana vitrectomy, scleral debridement, corneal patch graft, and amniotic membrane transplant were performed. The culture of vitreous fluid revealed *S. aurantiacum* and, thus, oral voriconazole was added to the therapeutical regimen. One month later, the patient experienced severe pain, which was resolved by another scleral debridement. The MRI image repeatedly demonstrated scleritis adjacent to the optic nerve. Because of a risk for other complications, which, in this case included even meningitis, the enucleation was performed.

The last case reported so far was described by Carnovale et al. in Argentina (1). A 4-year-old healthy girl was admitted to a hospital following surgery on her left eye for corneal trauma. Postoperative treatment consisted of topical and intravenous antibiotics and prednisolone. Ten days later, infectious endophthalmitis developed and was presumed to be likely caused by a mycotic agent due to the previous antibiotic therapy. A vitrectomy of the left eye was performed, and intravitreal injections of amphotericin B and vancomycin were administered. The result of a direct microscopic examination of the vitreous humor was negative. However, culture yielded *S. aurantiacum* after 15 days of incubation. Following the identification of *S. aurantiacum*, antifungal therapy was changed to voriconazole. The patient had a favorable clinical outcome, and no complications were reported.

*S. aurantiacum* was first described in 2005 and predominantly reported from the environment in Australia, but also in Japan and some European regions, including agricultural areas in the west of France, Netherlands, Spain, Germany, Italy, Latvia, and the United Kingdom (15–17). *S. aurantiacum* differs in the culture from the other species of this genus by its ability to grow in temperatures up to 45 °C. Another distinctive feature of this species is the production of a yellow diffusible pigment after a few days of incubation. Colonies of this species are dense, cottony to lanose, often concentric with aerial mycelium. The colors of the colonies range from yellow to gray or brown (11). In the microscopical examination, *S. aurantiacum* forms solitary conidiophores on aerial mycelium. The conidia are smooth and thick-walled and obovoid or subcylindrical. The identification of the species of *Scedosporium* may be complicated due to the similarities in the morphology. MALDI-TOF MS represents an optimal, reliable, and rapid diagnostic tool. The process of confirmation of the pathogen is preferably performed by the sequencing of a specific genome region, for example the ITS region (1). *S. aurantiacum* potentially represents an emerging pathogen for ocular infections, which is reportedly less susceptible to antifungals (based on the MIC values) than *S. apiospermum* (4). A cornerstone of the diagnosis of scedosporiosis remains to be a classical culture, despite the slower growth of this fungus, which generally takes 2–7 days. The rapid identification of *S. aurantiacum* can then be performed from well-grown colonies after colony pretreatment using MALDI-TOF MS or directly from the clinical specimen by real-time PCR detection (18). However, this species is so rarely involved in eye infections that probably the majority of laboratories will not perform PCR directly from the specimen. For species-level confirmation, sequencing of the ITS region and/or the *β*-tubulin gene is recommended (19). To conclude, all positive cultures from otherwise sterile sites have to be considered clinically significant (11).

In this study, a rare case of ocular infection caused by an emerging pathogen *S. aurantiacum* was described. Our case demonstrates a severe course of *S. aurantiacum* infection with unfavorable prognosis, where the inflammatory process in the eye could not be controlled. After 7 days of voriconazole treatment, the ocular complication with intractable pain claimed evisceration. Although fungal keratitis was diagnosed within two days of admission and *S. aurantiacum* was identified within three days using MALDI-TOF MS, the overall diagnostic process was delayed because the patient was referred to our hospital as late as seven days after the corneal ulcer had been recognized at another institution. A delayed diagnosis together with the patient's partial lateral tarsorrhaphy of the right eyelids and diabetes mellitus could play a significant role in the poor outcome of the treatment. However, the unfavorable course of the infection may also have been associated with the pathogenic potential of the *S. aurantiacum* and with the severity of infection. Only three such cases have been previously reported and well described in the literature. Out of the four cases in total, three had to be solved by enucleation or evisceration because of infectious complications. Only in the case of the young girl, the infectious endophthalmitis caused by corneal trauma was solved by voriconazole therapy without a need for surgical intervention. Of note, all four cases of ocular infection caused by *S. aurantiacum*, including in this study, occurred in female patients.

Out of the genus *Scedosporium*, *S. apiospermum* represents a relatively common pathogen in ocular infection. *S. aurantiacum* is likely more pathogenic, because it requires an extended treatment by voriconazole and, quite often, early surgical interventions (2). Therefore, the prognoses of ocular infections caused by *S. aurantiacum* seem to be usually worse. In the analysis performed by Cassano et al. (20), the authors reviewed ocular infections caused by *S. apiospermum*, of which 13 patients were managed primarily with antifungal therapy alone, sometimes combined with keratoplasty. In six others, the infection required enucleation. The potential factors of the virulence of *S. aurantiacum*, which may contribute to more complicated diseases, appear to be proteases (4). Interestingly, the pathogenicity of *S. aurantiacum* is considered to be higher than the pathogenicity of *S. apiospermum* and probably similar to that of *Lomentospora prolificans* (formerly *S. prolificans*), which is among the most virulent species in the genus *Scedosporium* and related genera (2, 21). Thus, correct identification to the level of the individual species and its antifungal susceptibility examination is crucial for the administration of the appropriate therapy, mainly by voriconazole. Although voriconazole is recommended for the treatment of scedosporiosis, these recommendations are primarily based on invasive infections, while evidence specific to *S. aurantiacum* remains extremely limited and is based on *in vitro* susceptibility testing (8).

Notably, Qiu et al. reported natamycin as a potential antifungal agent for the topical treatment of filamentous fungal keratitis (22). Furthermore, Khan et al. described the case of a patient with *Scedosporium* spp. keratitis successfully treated with topical natamycin, with complete resolution after four weeks of therapy (23). However, natamycin has several disadvantages, including its availability only as a topical formulation, limited corneal penetration, restricted availability in Europe, and a higher cost compared with voriconazole. Further studies are also needed to establish the efficacy of olorofim, a novel antifungal agent belonging to the new orotomide class, for the treatment of ocular infections caused by *S. aurantiacum*. Although olorofim has received FDA orphan drug designation in the United States, this designation is currently limited to the treatment of invasive scedosporiosis (24).

This study has several strengths. First, to the best of our knowledge, this represents the first reported case of keratitis caused by *S*. *aurantiacum* in the Czechia and potentially the first such case described in Europe. Second, MALDI-TOF MS enabled rapid identification, with one of three replicate measurements achieving a score consistent with species-level identification (>2.0), which is, according to the experience of our laboratory, not commonly achieved for rare filamentous fungi. The identification was further supported by the characteristic growth morphology and production of a yellow diffusible pigment. Finally, this case report expands the current knowledge of ocular infections caused by *S. aurantiacum* and may improve the management of similar cases in the future.

This study has also several limitations. First, sequencing of the 18S rRNA gene was unsuccessful and therefore could not be used to further support the identification of *S. aurantiacum*. In addition, the isolate was not submitted to the National Reference Laboratory for filamentous fungi, where strain preservation and further molecular characterization could have been performed. This was primarily attributable to the prolonged interval between the patient's presentation and the initiation of case report preparation. The second limitation is the lack of photographic documentation of the affected eye due to the rapidly progressive course of the disease.

In conclusion, we report a rare and probably the first European case of keratitis caused by *S. aurantiacum*. The diagnosis was established by culture and MALDI-TOF MS, supported by characteristic phenotypic findings. In our patient, the infection progressed despite antifungal treatment and required evisceration. Based on this single case, no clear conclusions can be drawn regarding the relative virulence of *S. aurantiacum*. However, together with the limited number of previously published cases, our findings support the hypothesis that ocular infections caused by *S. aurantiacum* may be associated with an aggressive clinical course and a frequent need for surgical intervention. Larger case series are required to clarify the pathogenic potential of this species and to define optimal therapeutic approaches.

## Data Availability

The raw data supporting the conclusions of this article will be made available by the authors, without undue reservation.
